# Aftermath histopathological findings of 2019 novel Coronavirus (COVID-19) pneumonia

**DOI:** 10.22088/cjim.13.0.303

**Published:** 2022

**Authors:** Mohammad Ranaei, Seyed Sina Taheri, Fatemeh Rojaei, Faezeh Zabihi

**Affiliations:** 1 Department of Pathology, Babol University of Medical Sciences, Babol, Iran; 2 Babol University of Medical Sciences, Babol, Iran

**Keywords:** Covid-19 pneumonia, novel coronavirus, pathology

## Abstract

**Background:**

There have been several studies describing clinicoradiological features of the novel coronavirus SARS-CoV-2 infection. It seems that we still should know more about pathological features in the different stages of this infection.

**Case presentation:**

A 77 year-old man with cough and respiratory distress was admitted to the intensive care unit.  Real-time PCR on nasopharyngeal swab was done for him and it was positive for SARS-CoV-2.He was treated with oxygen therapy, hydroxychloroquine and antibiotic therapy and was discharged from the hospital with brief improvement of clinical symptoms. However, due to persistent dyspnea, the patient was admitted to the hospital again and throracotomy and wedge biopsy were performed for about 3 months from the onset of symptoms.

**Conclusion:**

Pathological examination revealed diffuse alveolar damage, fibroblastic hyperplasia, infiltration of inflammatory cells and hyaline membrane formation.

Vaccination is one of the effective efforts of global scientific community to end the novel 2019 Novel coronavirus 2019 (COVID-19) pandemic. To date, the World Health Organization (WHO) has approved eight vaccines for emergency use: Pfizer/BioNTech, Moderna, AstraZeneca, Johnson and Johnson, Covishield, Sinovac, Sinopharm, and Bharat Biotech (1). Among these, the AstraZeneca vaccine, undergoing 47 clinical trials in 23 countries, has been recognized as one of the most effective, efficient, and safe vaccines, which has been approved and used by 124 countries so far (2). Since the administration of the vaccine, an immune-mediated thrombotic thrombocytopenia syndrome has been reported in several individuals worldwide (3-6). Quired thrombotic thrombocytopenic purpura (aTTP) is a rare hematologic disorder characterized by thrombotic microangiopathies causing multi-organ ischemia, including the cerebrum mainly and to lesser extent renal and cardia. This report documented the case of aTTP diagnosed after administration of AZD1222 Vaxzevria (AstraZeneca) vaccine. 

## Case presentation

 A 77-year old man was evaluated in our hospital because of severe respiratory distress and cough. He was an active smoker (about 1 pack year). There was a slight fine crackle on auscultation on the right lower lobe. Nasopharyngeal swab was done for him and it was positive for COVID-19 with cycle threshold (Ct) number 18. He was treated by assisted oxygenation, hydroxychloroquine and antibiotics. 

**Figure 1:**
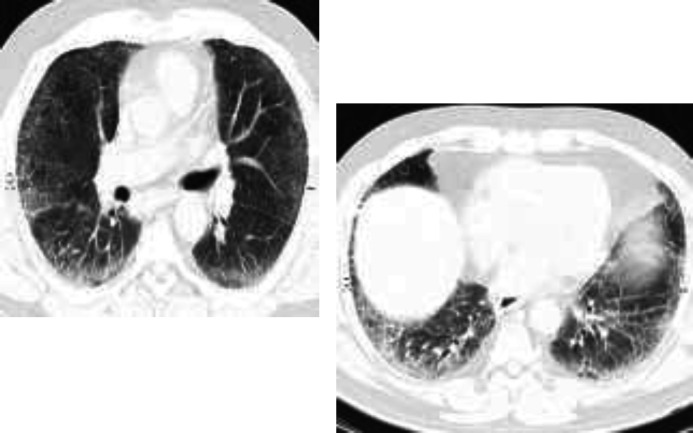
CT scan showing bilateral ground glass opacity and bronchiectasis

**Fig.2:**
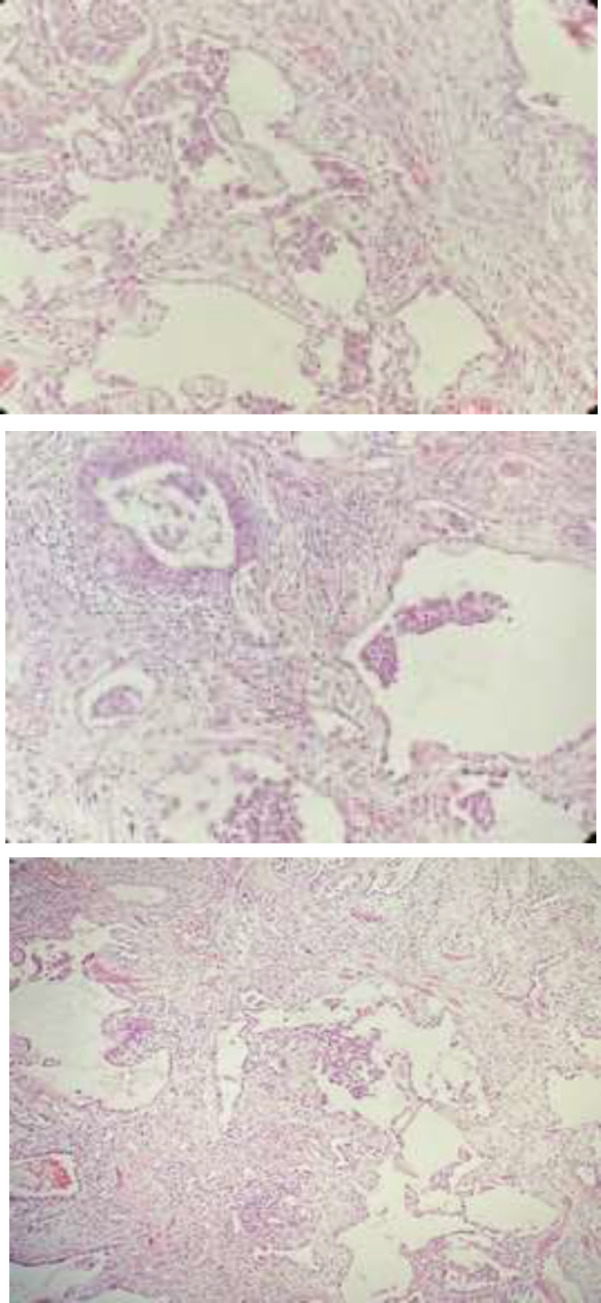
Sections reveal dilated lung alveolae, type II pneumocytes hyperplasia. intraalveolar hemorrahge, foamy macrophages, cellular or proteinaceous exudate accompanied by interstitial fibroblastic proliferation and fibrin deposition

His symptoms mildly decreased and recovered from the acute illness .Patient was not dependent on nasal oxygen and due to the time of peak of the SARS-CoV-2 infection and large number of critically ill patients in need of hospitalization; we decided to discharge our patient by symptomatic and conservative outpatient therapy at home, including famotidine, aspirin, atorvastatin. but he was admitted to the hospital again because of progressive orthopnea and desaturate on exercise. On clinical examination, he was suffering and dyspneic. His laboratory findings were: White blood cells (( WBC:7900/mm3) ;Hb:14/5 g/dl;platelets:165000/mm3; Lactic acid dehydrogenase ((LDH:754 unit/liter). CT scan was done and revealed bilateral and diffuse patchy ground glass opacity and lower lung bronchiectasis (Fig. 1). Because of lack of recovery from disease, progressive orthopnea and desaturate on exercise, The clinician with the patient‘s consent, decided to perform lung biopsy to clarify if any other added histopathology. Patient underwent throracotomy and wedge biopsy for about 3 months from the onset of first symptoms. Tissue specimen was fixed in 10% formalin for 48 h and then hematoxylin and eosin stain was performed. Sections were analyzed and revealed dilated lung alveolae lined by hyperplastic type II pneumocytes with enlarged and mild atypical nuclei and granular and clear cytoplasm. Intraalveolar hemorrahge, foamy macrophages, cellular or proteinaceous exudate. Desquamation of epithelial cells, hyaline membrane formation and intra alveolar thickening were also seen. Interstitial fibroblastic proliferation and fibrin deposition were evident (fig.2)**. **

**Patient outcome:** his symptoms moderately improved but not completely, and he is still suffering from exercise dyspnea.

## Discussion

The COVID-19 pandemic, also known as the coronavirus, is an ongoing pandemic of coronavirus disease 2019 (COVID-19) caused by severe acute respiratory syndrome coronavirus 2 (SARS-CoV-2), which is RNA virus. It has high variable symptoms ranging from none to severe respiratory distress and other organs failure. The standard method of testing for the presence of SARS-CoV-2 is real-time reverse transcription polymerase chain reaction (rRT-PCR) which detects the presence of viral RNA fragments. The test is typically done on respiratory samples obtained by a nasopharyngeal swab. Lung biopsy has limited value for disease diagnosis, but better identification of histopathological features is necessary. Most of the patients experience long recovery including prolonged respiratory distress, desaturate on exercise and anorexia. So we evaluated a symptomatic covid 19 infected patient (that was proven by PCR test) because of his remaining respiratory symptoms for about 3 months. We analyzed his pathological specimens which revealed fibroblastic proliferation, fibrin and hyaline deposition, viral cytopathic effect and pneymocyte II hyperplasia. These findings are suggestive for diffuse alveolar damage in organizing phase. Aliofi et al. (1) presented 2 cases with covid-19 infection and severe respiratory distress which underwent blebectomy and pleurectomy on day 16^th^ and 23^rd^ after symptom onset. They declared non pathogonomonic findings such as alveolar damage with septa disruption, desquamation, edema, and proteinaceous exudates and alveolar congestion. Diffused peripheral vessels endothelial hyperplasia, muscular wall thickening, and intravascular hemorrhagic thrombosis were also declared as late histopathological findings of those two cases. Tian et al. (2) evaluated 2 known cases of lung adenocarcinoma with superimposed early phase of covid-19 infection. Pathologic findings from these two patients were edema and prominent proteinaceous exudates, vascular congestion, and inflammatory clusters with fibrinoid material and multinucleated giant cells. Reactive alveolar epithelial hyperplasia and fibroblastic proliferation (fibroblast plugs) were also seen. In a cohort study, Borczuk et al. (3) examined the pathological findings of 86 autopsy cases, which revealed the frequent presence of tracheobronchial inflammation, histologic features of DAD at different stages, chronic interstitial inflammation, and vascular injury

Our findings revealed late lung histopathological findings of a symptomatic Covid-19 infected patient including diffuse alveolar damage in organizing phase. Now we are in global pandemic SARS-CoV-2 infection and many infected patients were not evaluated in the early phase of the disease, so we suggest that we should consider Covid-19 infection for every patient with unknown chronic lung disease especially the patients who has diffuse alveolar damage in lung biopsy. Further studies are needed on more infected patients with prolonged respiratory symptoms that could be beneficial for the better planning of patients’ follow-up and treatment.
